# Successful Prevention of Fetal Autoimmune-Mediated Heart Block by Combined Therapies With Hydroxychloroquine and Intravenous Immunoglobulin: A Case Report

**DOI:** 10.3389/fcvm.2021.759260

**Published:** 2021-11-12

**Authors:** Li Zhao, Yan Zhou, Chuan Wang, Yifei Li, Qi Zhu, Yimin Hua, Lina Qiao, Jinlin Wu, Kaiyu Zhou

**Affiliations:** ^1^Key Laboratory of Birth Defects and Related Diseases of Women and Children of Ministry of Education, Department of Pediatrics, West China Second University Hospital, Sichuan University, Chengdu, China; ^2^Respiratory Department of Pengzhou Hospital of Traditional Chinese Medicine, Chengdu, China; ^3^Department of Ultrasonaography, West China Second University Hospital, Sichuan University, Chengdu, China

**Keywords:** fetal heart, atrioventricular block, autoantibody, prophylactic therapy, intravenous immunoglobulin

## Abstract

A fetal autoimmune-mediated atrioventricular block is a passively acquired autoimmune disease in which maternal autoantibodies enter the fetal circulation via the placenta and subsequently cause inflammation and fibrosis of the atrioventricular node. Once fetal autoimmune-mediated atrioventricular block occurs, it only takes a short time to progress from first-degree atrioventricular block to complete atrioventricular block, meaning that the damage is often irreversible. Autoimmune—associated AVB, a rare but life—threatening disorder, occurs in 2–5% of pregnancies with positive anti—Ro/SSA (the most common one) and La/SSB antibodies. The perinatal mortality of neonates with AVB outlined in research is approximately 30%. Thus far, for autoimmune-associated AVB fetuses, currently used treatments include corticosteroids, hydroxychloroquine, intravenous immunoglobulin (IVIG), b—sympathomimetic agent, and even plasma exchange. Currently, approaches for preventing the progression and recurrence of a fetal atrioventricular block are still controversial. Here, we reported a baby of successful prevention from the fate of the fetal atrioventricular block by adopting prophylactic comprehensive prenatal therapy.

## Introduction

Fetal autoimmune-mediated atrioventricular block (AVB) is defined as a type of disease in which maternal connective tissue disease is confirmed (including Sjogren's syndrome, systemic lupus erythematosus, et al.) or it can be a case with asymptomatic positive autoantibodies, meaning that the fetus develops AVB without any cardiac structural abnormality (1). Maternal serum autoantibodies mainly include anti-SSA/Ro and anti-SSB/La antibodies (2). SSA/Ro antigen is a ribonucleoprotein complex that comprises Ro52 and Ro60 polypeptides. Existing studies have found that the main antibody causing fetal autoimmune—associated AVB is anti-Ro52 antibody (95%), followed by anti-Ro60 (63%) and anti-La antibody (58%) (3). At present, the pathogenesis of fetal autoimmune—associated AVB is thought to mainly include inflammation and electrophysiology. Ambrosi and Wahren-Herlenius (4) proposed the apoptosis hypothesis and the cross-reactivity hypothesis. In the apoptosis-inflammatory hypothesis, the maternal autoantibodies (mainly anti-Ro60 antibodies) are transported into the fetal circulation through the placenta and may bind to apoptotic cardiac cells during apoptosis normally occurring in the developing fetal heart and thus divert the removal of apoptotic debris from a non-inflammatory pathway to engulfment by macrophages through opsonization, inducing the release of inflammatory factors, triggering a series of reactions to promote inflammation and fibrosis. Additionally, the cross-reactivity hypothesis holds that maternal autoantibodies inhibit the activation of calcium channels in myocytes, resulting in intracellular calcium homeostasis imbalance, thereby affecting action potential spread and atrioventricular junction conduction. Since first-degree AVB could rapidly progress to second- even the third-degree AVB, which is also known as complete AVB (CAVB), more than 60% of fetuses have progressed to CAVB by the time discovered. Once a complete heart block occurs, the damage is largely irreversible (5), and the intrauterine and neonatal mortality rates are significantly increased, with more than two-thirds of the surviving children requiring permanent pacemakers (6). Therefore, early diagnosis and proper intervention of low-degree AVB in fetuses are of vital importance to their quality of life and long-term prognosis. Previous studies have shown that transplacental administration of corticosteroids should be considered for fetuses diagnosed with first-degree AVB. If it advances to second-degree AVB and CAVB, in addition to corticosteroids therapy, combined hydroxychloroquine, IVIG and plasma exchange therapy can be considered. The mechanism of hydroxychloroquine and dexamethasone action may directly regulate the autoimmune-mediated inflammation of the fetal heart by reducing the level of maternal antibodies and translocating through the placenta, thereby preventing the immune damage of the fetal heart. And IVIG may play a therapeutic role by binding to Fc receptors on the surface of macrophages to inhibit the progression of inflammatory response. However, there is no effective and unified treatment strategy for the prevention and/or improvement of fetal autoimmune—associated AVB due to the relatively low incidence (7). Herein, we report a case of a pregnant woman who promptly received prophylactic therapy with intravenous IVIG with early suspected fetal autoimmune—associated AVB, and finally successfully delivered a healthy baby.

## Ethics Statement

Informed written consent was obtained from the patient with full, comprehensive, and detailed communication with our Fetal Cardiac therapeutic Team. The patient has provided informed consent for the publication of this case.

## Case Presentation

A 28-year-old pregnant woman, 12 gestational weeks in July 2019, attracted our attention for recurrent abortions, as well as being strongly positive of anti-SSA /Ro and antinuclear antibodies, suspected fetal autoimmune associated AVB without cardiac structural abnormality.

The first pregnancy of this woman was in March 2017 but it resulted in a spontaneous abortion at 50+ days of pregnancy. Her second pregnancy in February 2018 was a biochemical pregnancy. Subsequently, a thyroid function test suggested hypothyroidism and was successfully treated by oral thyroxine. Whereas, strongly positive antinuclear antibody (granular type) with a titer of 1:1,000 (normal reference value is negative) and anti-SSA/Ro antibody with a quantitative >400 RU/ml (normal reference range 0 to <20 RU/ml) of the patient was revealed and was diagnosed of undifferentiated connective tissue disease by entomologist, and was given hydroxychloroquine (200 mg, twice a day), tacrolimus (500 mg, twice a day), and low-dose methylprednisolone (4 mg, once a day). Except for repeated abortions, the patient had not complained of photosensitive rash, thrombosis, joint pain, and arthritis, Raynaud's phenomenon, xerophthalmia, or xerostomia.

The patient was pregnant the third time in July 2019, and the re-examination of autoantibodies revealed antinuclear antibody-positive (particle type) with a titer of 1:320; anti-SSA/Ro antibody-positive with a quantitative >400 RU/ml. As the pregnancy progressed, monitoring the patient's serum antibody revealed that the anti-Sm antibody gradually became positive (>400 RU/ml, the normal reference range 0 to <20 RU/ml), but there was still no clinical sign of connective tissue disease. Hydroxychloroquine (200 mg, twice a day), tacrolimus (500 mg, twice a day), and low-dose methylprednisolone (4 mg, once a day) continued.

The patient was transferred to our hospital due to recurrent abortion and undifferentiated connective tissue disease by a local obstetrician and endocrinologist. Considering that the first two pregnancies of the patient were both early miscarriages, it is still uncertain whether there exited fetal immune heart injury in the first two pregnancies, and they might be probands of fetal immune heart damage to this pregnancy. Existing epidemiological studies suggest that the risk of fetal AVB will be significantly increased in a pregnancy with a previously affected fetus or neonate, and the role of fetal genetic susceptibility in the development of autoimmune—associated fetal AVB cannot be ignored. Therefore, we classified this pregnancy as a high-risk pregnancy of cardiac immune attack (7). During the whole pregnancy, the fetal AV interval and maternal serum autoantibody titers were regularly monitored. The mother was generally in good physical condition during pregnancy and no abnormal findings in the ECG and echocardiogram examinations.

At 12 weeks of gestation, we monitored the fetal echocardiogram and found that there exited strong light points about 2 mm in diameter in the left ventricular, as well as enhanced endocardial echo image, which indicated that the fetal heart might exit a certain degree of immune injury. At the same time, the fetal echocardiogram monitoring indicated that the AV interval was within the normal range (104–112 ms). Considering the relatively high incidence of fetal heart immune-mediated cardiac conduction injury in pregnancies that suffer from immune-mediated fetal congenital heart block (1, 8, 9), avoiding the occurrence of miscarriage or stillbirth, and potential fetal AVB, dexamethasone and intravenous immunoglobulin (IVIG) were treatment options according to the treatment regimens reported in previous literature (10–13).

After fully understanding the advantages and disadvantages of dexamethasone and IVIG, the patient determined to adopt IVIG treatment (Chengdu Rongsheng Pharmaceutical Co., LTD., 2.5 g, 50 ml/bottle). According to the characteristics of IVIG, and the reported usage in the literature (14–16), the patient received IVIG 1 g/kg twice a week at 12 and 13-week of pregnancy, respectively. Encouragingly, in a reexamination of the fetal echocardiogram, the enhanced echoes and endocardial echo enhancement of the left ventricle gradually disappeared, and the AV interval was normal without a tendency of prolongation. Meanwhile, the maternal serum antibody titers showed a downward trend after receiving IVIG (Table 1).

**Table 1:** Monitoring of the fetal HR, AV interval, maternal serum antibody and medication during pregnancy.

**Gestational weeks**	**Heart rate(bpm)**	**AV interval (ms)**	**Anti-SSA/Ro antibodies**	**Antinuclear antibodies (titer)**	**Usage of IVIG**
Prior to conception	—	—	>400 RU/ml	1:1,000	—
12 + 1 weeks	149	108	>400 RU/ml	1:320	Twice a week
13 + 1 weeks	151	104	—	—	Ibid
14 + 6 weeks	153	100	—	—	Once two weeks
16 + 6 weeks	150	105	>400 RU/ml	1:1,000	Ibid
20 + 6 weeks	146	113	—	–	Once a month
24 + 6 weeks	148	120	>400 RU/ml	1:1,000	Ibid
25 + 6 weeks	143	138	—	—	Once every 3 weeks
27 + 6 weeks	146	130	>400 RU/ml	1:1,000	Ibid
30 + 6 weeks	142	123	—	—	Ibid
33 + 6 weeks	140	123	—	—	Ibid (the last dosage)
After delivery	—	—	282.2 RU/ml	1:320	—

*AV, atrioventricular; ibid, ibidem (in the same location)*.

**Figure 1:**
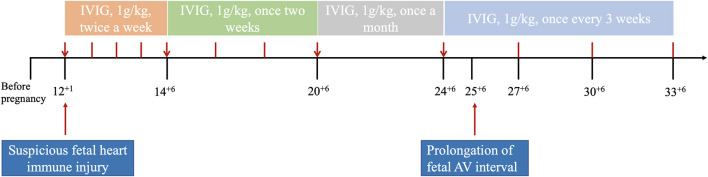
The patient was treated with methylprednisolone (8 mg, once a day), hydroxychloroquine (200 mg, twice a day), and tacrolimus (2 mg, twice a day) throughout this pregnancy. And important events during pregnancy are indicated by long red arrow symbols. Adjustment time-point of IVIG are marked by short red arrows, and the frequency of usage are marked by red bars.

Then, the frequency of IVIG treatment was adjusted to once every 2 weeks, 1 month later, regarding fetal stable condition, and then adjusted once a month. However, at 25 + 6 weeks of gestation, a gradual prolongation of the AV interval was observed, increasing from 106 ms to 130–140 ms. Although the AV interval was still within the normal range, the rapidly extending within 1 week was urgent. In order to avoid progressive prolongation of the fetal AV interval, we upregulated the frequency of IVIG treatment to once every 3 weeks based on the half-life of immunoglobulin in human circulation (21 days). After adjusting the frequency of IVIG treatment, the AV interval of the fetal heart gradually returned to the previous condition. The last IVIG dosage was given at 33 + 6 GW. The fetal condition during pregnancy and maternal serum antibody level are shown in Table 1, and the use of IVIG is shown in Figure 1.

Fetal echocardiography was regularly performed until successful delivery, and there were no signs of fetal arrhythmia and/or cardiac malformations. Cesarean section was performed at 39 weeks of gestation and a male newborn was born with a birth weight of 2,940 g. The amniotic fluid was clear at birth, the Apgar score was 10 points, and there were no abnormalities in the placenta or expectation.

The newborn underwent a comprehensive physical examination after delivery and received blood routine tests, thyroid function tests, and echocardiography examination 3 days after being born, all showed within normal range. The electrocardiogram of the newborns was also performed on the first day after birth, and the results were normal with a PR interval of 104 ms, as well as the Holter monitoring. Meanwhile, the serum autoantibody examination of the baby showed positive results of anti-Sm antibodies (quantitative >400 RU/ml), anti-SSA/Ro antibodies (quantitative 148.7 RU/ml), and antinuclear antibodies (titer 1:100).

At 1-, 3-, 6-, 12-, 18-month follow-ups, the growth and development, ECG, and echocardiography of the baby were normal. Additionally, all the serum antibody titers also turned negative at 3- or 6-month of follow-up (Table 2).

**Table 2:** Follow-up of the baby after birth.

**Follow-up time**	**Height (cm)**	**Weight (kg)**	**Anti-SSA/Ro antibodies**	**Antinuclear antibodies (titer)**	**Anti-Sm antibodies**
At birth	50	2.94	148.7 RU/ml	1:100	>400 RU/ml
1st-month	54	4.15	—	—	—
3rd-month	60	6.50	32.3 RU/ml	Negative	Negative
6th-month	68	8.20	Negative	Negative	Negative
12th-month	76	9.60	Negative	Negative	Negative
18th-month	82	11.5	Negative	Negative	Negative

During the post-partum period, the mother remained asymptomatic and showed no signs of aggravation of the disease. The monitoring of maternal serum autoantibody level within 6-month follow-up indicated a gradual decline. While the recent laboratory tests showed that anti-Sm antibodies with quantitative >400 RU/ml, anti-SSA /Ro antibodies with quantitative 282.2 RU/ml, and antinuclear antibodies with titrum 1:320.

## Discussion

Fetal autoimmune—associated AVB, of which anti-Ro/SSA (the most common one) antibodies and anti-La/SSB antibodies are the main pathogenic antibodies, is the primary cause of congenital AVB, accounting for 60–90% of the fetal AVB. The risk of developing a fetal AVB varies between 2 and 5% in pregnancies with positive anti—Ro/SSA and La/SSB antibodies and increases to 12–25% in a pregnancy with a previously affected fetus or neonate (1, 7, 8, 13). Therefore, proper prophylactic therapy could be justified in the latter group to reduce fetal intrauterine mortality and improve prognosis.

Regarding the present management of autoimmune—mediated fetal AVB, the methods mainly include corticosteroids, immunoglobulins, plasma exchange, etc. However, the efficacy of current approaches for preventing the progression and recurrence of fetal AVB is still controversial. Pisoni et al. (16) and Friedman et al. (15) found that low doses of IVIG were not effective as prophylactic therapy for preventing fetal AVB in high-risk pregnancies. This finding is consistent with the study of Friedman et al. On the contrary, some studies have suggested that IVIG could prevent the progress of immune-mediated fetal AVB by reducing the maternal serum antibody titer (12, 17). The murine model of Kaaja and Julkunen (18) also provides evidence that maternal administration of IVIG inhibits the transfer of potentially pathogenic anti-Ro/La antibodies across the placenta and their subsequent deposition in the fetal heart. These studies provide a rationale for the anti-inflammatory effect of IVIG in preventing the recurrence of fetal AVB.

In our case, the two previous miscarriages of the mother significantly increased the risk of developing fetal AVB during this pregnancy. And through the monitoring of fetal echocardiography in early pregnancy, it was found that the fetal heart enhanced echo and endocardial hyperactivity, which indicated that the fetal heart may have an immune injury. As a result, the patient received prophylactic therapy with IVIG, and subsequently, the fetal heart abnormal changes gradually disappeared in fetal echocardiography monitoring after treatment. We supposed that these echocardiographic changes meant that the immune inflammatory response of the fetal heart was partially controlled. In addition, although fetal PR interval monitored during pregnancy showed a trend of gradually prolonging, it also gradually recovered to the previous level after adjusting the frequency of IVIG administration, which also suggests that the fetal heart immune injury may be controlled to a certain extent.

Previous studies have revealed that the average time for seroconversion of serum antibodies during pregnancy in newborns without IVIG treatment is 1–2 years after birth. However, this baby's serum antibodies turned negative at follow-up 6 months after birth. During the postnatal follow-up to 1 year old, no cardiac conduction block, structural and functional abnormalities were found by both electrocardiogram and echocardiography. The growth and development were normal and no neuropathologic symptoms and/or signs were observed. The most encouraging thing was this baby avoided the fate of postnatal cardiac pacing treatment through preventive treatment. Furthermore, no adverse reactions were detected during IVIG treatment in the mother, and the level of serum autoantibody levels decreased rapidly in postpartum monitoring, indicating the risks of long-term exposure of vital organs to positive autoantibodies was minimized. It not only successfully prevented the occurrence of fetal AVB but also reduced the maternal serum antibody titer, which exhibited potential benefits to maternal health.

Therefore, regarding the current absence of standard and recommended IVIG treatment options, the IVIG prophylactic treatment used in our case might be a potential recommendation for pregnant patients with this high-risk factor of fetal AVB when the suspected fetal cardiac immune injury occurs. Moreover, there is no unified strategy for the initial time, frequency, and dosage of IVIG treatment for immune-mediated fetal heart injury. Further clinical research is needed to establish a standardized protocol. Our experience from this single case of high dose of IVIG from first to third trimester might provide a source for further therapeutic exploration, and may even provide therapeutic guidance for some special or severe cases after weighing the risks and benefits.

## Data Availability Statement

The original contributions presented in the study are included in the article/supplementary material, further inquiries can be directed to the corresponding author/s.

## Ethics Statement

Written informed consent was obtained from the individual(s), and minor(s)' legal guardian/next of kin, for the publication of any potentially identifiable images or data included in this article.

## Author Contributions

KZ and LQ were responsible for the study design and manuscript preparation. YZ, LQ, CW, QZ, and KZ were responsible for the clinical data. YL and YZ contributed to data acquisition. LZ and KZ wrote this manuscript. JW, LZ, and KZ contributed to revision of this manuscript. All authors read and approved the final manuscript.

## Funding

This work was supported by key R&D projects of the Sichuan Science and Technology Department (Nos. 2020YFS0101, 2021YJ0211, and 2021YFS0094).

## Conflict of Interest

The authors declare that the research was conducted in the absence of any commercial or financial relationships that could be construed as a potential conflict of interest.

## Publisher's Note

All claims expressed in this article are solely those of the authors and do not necessarily represent those of their affiliated organizations, or those of the publisher, the editors and the reviewers. Any product that may be evaluated in this article, or claim that may be made by its manufacturer, is not guaranteed or endorsed by the publisher.
